# The majority of severe COVID-19 patients develop anti-cardiac autoantibodies

**DOI:** 10.1007/s11357-022-00649-6

**Published:** 2022-09-16

**Authors:** Miklós Fagyas, Béla Nagy, Arnold Péter Ráduly, Ivetta Siket Mányiné, Lilla Mártha, Gábor Erdősi, Sándor Sipka, Enikő Enyedi, Attila Ádám Szabó, Zsófia Pólik, János Kappelmayer, Zoltán Papp, Attila Borbély, Tamás Szabó, József Balla, György Balla, Péter Bai, Attila Bácsi, Attila Tóth

**Affiliations:** 1 Division of Clinical Physiology, Department of Cardiology, Faculty of Medicine, University of Debrecen, Debrecen, Hungary; 2 Division of Cardiology, Department of Cardiology, Faculty of Medicine, University of Debrecen, Debrecen, Hungary; 3 HAS-UD Vascular Biology and Myocardial Pathophysiology Research Group, Hungarian Academy of Sciences, Budapest, Hungary; 4 Research Center for Molecular Medicine, Faculty of Medicine, University of Debrecen, Debrecen, Hungary; 5 Department of Laboratory Medicine, Faculty of Medicine, University of Debrecen, Debrecen, Hungary; 6 Kálmán Laki Doctoral School, University of Debrecen, Debrecen, Hungary; 7 Department of Pediatrics, Faculty of Medicine, University of Debrecen, Debrecen, Hungary; 8 Department of Internal Medicine, Faculty of Medicine, University of Debrecen, Debrecen, Hungary; 9 Department of Medical Chemistry, Faculty of Medicine, University of Debrecen, Debrecen, Hungary; 10 MTA-DE Lendület Laboratory of Cellular Metabolism, Debrecen, Hungary; 11 MTA-DE Cell Biology and Signaling Research Group ELKH, Debrecen, Hungary; 12 ELKH-DE Allergology Research Group, Debrecen, Hungary; 13 Department of Immunology, Faculty of Medicine, University of Debrecen, Debrecen, Hungary

**Keywords:** COVID-19, Anti-cardiac autoantibodies, SARS-CoV-2

## Abstract

Severe cases of COVID-19 are characterized by an inflammatory burst, which is accompanied by multiorgan failure. The elderly population has higher risk for severe or fatal outcome for COVID-19. Inflammatory mediators facilitate the immune system to combat viral infection by producing antibodies against viral antigens. Several studies reported that the pro-inflammatory state and tissue damage in COVID-19 also promotes autoimmunity by autoantibody generation. We hypothesized that a subset of these autoantibodies targets cardiac antigens. Here we aimed to detect anti-cardiac autoantibodies in severe COVID-19 patients during hospitalization. For this purpose, 104 COVID-19 patients were recruited, while 40 heart failure patients with dilated cardiomyopathy and 20 patients with severe aortic stenosis served as controls. Patients were tested for anti-cardiac autoantibodies, using human heart homogenate as a bait. Follow-up samples were available in 29 COVID-19 patients. Anti-cardiac autoantibodies were detected in 68% (71 out of 104) of severe COVID-19 patients. Overall, 39% of COVID-19 patients had anti-cardiac IgG autoantibodies, while 51% had anti-cardiac autoantibodies of IgM isotype. Both IgG and IgM anti-cardiac autoantibodies were observed in 22% of cases, and multiple cardiac antigens were targeted in 38% of COVID-19 patients. These anti-cardiac autoantibodies targeted a diverse set of myocardial proteins, without apparent selectivity. As controls, heart failure patients (with dilated cardiomyopathy) had similar occurrence of IgG (45%, *p* = 0.57) autoantibodies, while significantly lower occurrence of IgM autoantibodies (30%, *p* = 0.03). Patients with advanced aortic stenosis had significantly lower number of both IgG (11%, *p* = 0.03) and IgM (10%, *p* < 0.01) type anti-cardiac autoantibodies than that in COVID-19 patients. Furthermore, we detected changes in the anti-cardiac autoantibody profile in 7 COVID-19 patients during hospital treatment. Surprisingly, the presence of these anti-cardiac autoantibodies did not affect the clinical outcome and the prevalence of the autoantibodies did not differ between the elderly (over 65 years) and the patients younger than 65 years of age. Our results demonstrate that the majority of hospitalized COVID-19 patients produce novel anti-cardiac IgM autoantibodies. COVID-19 also reactivates resident IgG autoantibodies. These autoantibodies may promote autoimmune reactions, which can complicate post-COVID recuperation, contributing to post-acute sequelae of COVID-19 (long COVID).

**Supplementary Information:**

The online version contains supplementary material available at 10.1007/s11357-022-00649-6.

## Introduction

Severe acute respiratory syndrome coronavirus 2 (SARS-CoV-2) is responsible for the global coronavirus disease 2019 (COVID-19) pandemic, resulting in millions of deaths worldwide and impacting our everyday life. COVID-19 disproportionately affects the aged population [1]⁠. COVID-19 apparently has two phases. The acute phase (7–10 days for mild cases, 3–6 weeks for severe cases) is responsible for most of the observed mortality [2, 3]⁠. COVID-19 patients tend to also suffer from chronic symptoms, after resolution of the acute disease, called long COVID (also referred as post-COVID syndrome) [3]⁠. Long COVID develops in the majority of severe COVID-19 patients (83% of the cases [4]⁠), while it appears to affect about 35% of post-COVID patients with mild acute symptoms [5]⁠. It is also important to note that although COVID-19 mortality is associated with pulmonary function, it does not only target the lungs. COVID-19 also evokes cardiovascular complications, including acute coronary syndromes and cardiomyopathy [6]⁠, besides to systemic inflammation (especially in children) [7]⁠.

Recently, it has been reported that SARS-CoV-2 infection causes development of autoantibodies in patients with severe COVID-19. A set of targets has been identified recently for autoantibodies, including rheumatoid disease-related antigens [7]⁠ and interferons [8]⁠, which the latter were also implicated in COVID-19 mortality [9]⁠ by suppressing immune responses. Nevertheless, it is likely that autoantibodies in COVID-19 may be also directed against other targets [10]⁠.

These previous reports suggest the production of autoantibodies against various targets in vital organs upon SARS-CoV-2 infection. Here we tested the appearance of anti-cardiac autoantibodies in the serum of severe COVID-19 patients (*n* = 104, having a mortality of 57,104 patients). More than two-thirds of COVID-19 patients harbored anti-cardiac autoantibodies. The majority of these immunoglobulins were IgM, representing novel autoantibody production.

## Methods

### Patient recruitment

In this study, 104 consecutive COVID-19 patients older than 18 years of age were recruited in parallel at two medical centers (Clinical Center and Gyula Kenézy Campus, University of Debrecen, Debrecen, Hungary, and National Institute of Hematology and Infectiology, South-Pest Central Hospital, Budapest, Hungary) as described earlier [11]⁠. In addition, heart failure patients with dilated cardiomyopathy (*n* = 40) and patients with severe aortic stenosis (*n* = 20) were recruited as controls. COVID-19 patients suffered from different degrees of acute respiratory distress at admission and were confirmed to be positive for SARS-CoV-2 infection by reverse transcription-polymerase chain reaction (RT-qPCR) test of a nasopharyngeal swab. Detailed patient’s data are provided in Table 1.

**Table 1:** Clinical characteristics of patients enrolled into the study

Clinical parameter	Patients with COVID-19	Patients with dilated cardiomyopathy (end-stage heart failure)	Patients with advanced aortic stenosis
Number of patients	104	40	20
Age (median (IQR)), *n*	65 (52.25–72), 104	55 (46–61), 40	80 (78–83)
Male/female	6737	355	119
IL-6 (ng/L, median (IQR)), *n*	82 (26–185), 104	N/A	N/A
CRP (mg/L, median (IQR)), *n*	157 (62–240), 104	N/A	N/A
Ferritin (median (IQR)), *n*	952 (557–1603), 97	N/A	N/A
GFR (ml/min/1.73 m^2^, median (IQR)), *n*	63 (42–83), 104	N/A	N/A
Hemoglobin (g/L, median (IQR)), *n*	134 (120–147), 104	134 (115.5–144), 40	N/A
White blood cell count (giga/L, median (IQR)), *n*	8.91 (6.663–11.80), 104	8.46 (6.515–10.24), 40	N/A
NT-proBNP (ng/L, median (IQR)), *n*	N/A	2848 (708–5382), 40	2776 (688–11,437), 8
Diabetes mellitus, *n*, %	36, 35	11, 28	4, 20
Hypertension, *n*, %	78, 75	23, 58	15, 75
COPD, *n*, %	14, 13	8, 20	5, 25
Atrial fibrillation, *n*, %	24, 23	20, 50	11, 55
Renal insufficiency, *n*, %	23, 22	7, 18	13, 65
Hypothyreosis, *n*, %	6, 6	1, 3	0, 0
Left ventricular end-diastolic diameter (mm, median (IQR)), *n*	N/A	68.5 (65–78.5), 40	58 (49.5–64.5), 17
Left ventricular end-systolic diameter (mm, median (IQR)), *n*	N/A	58.5 (51.5–65), 40	40 (34.5–48.5), 17
Ejection fraction (%, median (IQR)), *n*	N/A	26.5 (20–33), 40	47.5 (30.75–55.5), 20
Body mass index (kg/m^2^, median (IQR)), *n*	N/A	29.35 (27.45–33.16) 40	25.15 (22.49–30.06), 14
Aortic root area (cm^2^, median (IQR)), *n*	N/A	N/A	0.5 (0.4–0.5), 15
Maximal aortic flow velocity (m/s, median (IQR)), *n*	N/A	N/A	78 (54–99), 19

### Ethical approval

Ethical approvals were issued by the Scientific and Research Ethics Committee of the University of Debrecen and the Ministry of Human Capacities. Recruiting COVID-19 patients was approved under the registration number 32568-32020EÜIG [11]⁠. Recruiting patients with heart failure with dilated cardiomyopathy [12]⁠ or with aortic stenosis [13]⁠ was approved under the registration identifier UDCC RECIEC 4375–2015. The human heart samples used for the identification of the autoantibodies were collected and banked under the cover of an ethical permit issued by the Hungarian Ministry of Health (323–82,005-1018EKU).

### Blood sampling and laboratory analyses

Peripheral blood samples drawn within the first day of hospital admission (labelled by day 0 in the figures) and follow-up samples were also available before discharge or death in 29 COVID-19 subjects. Patient age, pre-existing comorbidity, COVID-19 specific treatment, administration of invasive ventilation, and the outcome were recorded by clinicians. Routinely available laboratory serum tests were performed at the Department of Laboratory Medicine, University of Debrecen. Sera were stored at − 70 °C until anti-cardiac autoantibody testing.

### Detection of anti-cardiac autoantibodies

Anti-cardiac autoantibodies were detected by a Western blot-based method (Fig. s1). Human heart homogenate was obtained from unused donor hearts obtained from multiorgan donors. Heart samples were kept at − 70 °C until the experiments. 50–80 mg of wet left ventricular tissue was solubilized in SDS-PAGE sample buffer (Merck KGaA, Darmstadt, Germany) using a potter device. Samples were boiled for 5 min at 100 °C and then centrifuged for 10 min at 10,000 g, and the supernatants were used for the experiments. Protein concentration of supernatants was measured on a dot blot-based method (1 µl of sample was dotted onto nitrocellulose membrane and stained by Coomassie brilliant blue (Merck KGaA, Darmstadt, Germany) and contrasted to a standard made of bovine serum albumin (Merck KGaA, Darmstadt, Germany)). Picture recording was done by a MF Chemibis 3.2 (DNR Bio-imaging Systems, Neve Yamin, Israel) and evaluated by ImageJ software [14]⁠. Calibration and estimation of protein concentration was done by GraphPad Prism software (GraphPad Software, San Diego, CA, USA). Eighty micrograms of heart homogenate was loaded onto SDS-PAGE gels in a volume of 20 µl. Proteins were separated on self-casted 10% polyacrylamide gels by a Bio Rad gel electrophoresis apparatus (Bio Rad Laboratories, Hercules, CA, USA). Proteins were transferred to nitrocellulose membrane (from Bio Rad). Membranes were then immersed into DPBS (137 mM NaCl, 2.7 mM KCl, 10 mM Na_2_HPO_4_, and 1.8 mM KH_2_PO_4_, all from Merck KGaA, Darmstadt, Germany). Nonspecific protein binding sites were blocked by 5% nonfat milk in DPBS. Membranes were then cut along the lines of bands and placed into separate chambers (see Fig. s1). Strips were probed with human serum samples at a dilution of 1:1,000 separately. Membrane strips were then washed in DPBS 3 times (5 min each) and then collected into a common chamber to be incubated with peroxidase conjugated anti-human IgG (dilution 1:25,000, developed against the full IgG, recognizing both heavy and light chains) or anti-human IgM (dilution 1:10,000, Cat No. SAB3701404). Antibodies were obtained from Merck KGaA (Darmstadt, Germany). Strips were then washed and incubated with enhanced chemiluminescence solution for 1 min and then fitted together resembling the original membrane in the tray of MF Chemibis 3.2 imaging device. Images were recorded and saved for further evaluation. Images were evaluated by three authors familiar with Western blot (MF, ZP, and AT), and anti-cardiac autoantibodies were accepted if all agreed. All recognized bands are labelled in the dataset available online https://figshare.coms792b7b1ca2e40f3f3c93 (IgG detection) and https://figshare.comsd0911650287cc2c96979 (IgM detection).

### Determination of the molecular weight of cardiac autoantigens

**Fig. 1:**
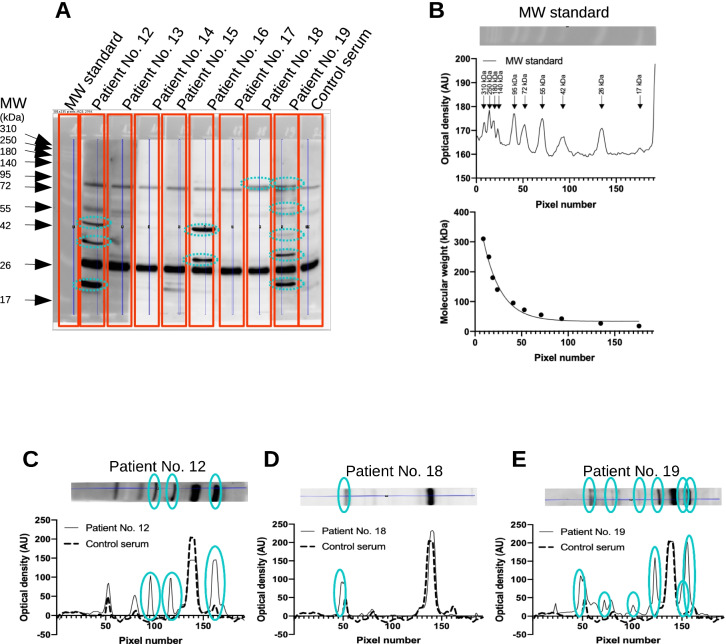
Detection of anti-cardiac autoantibodies in the sera of COVID-19 patients. Human heart homogenate was separated by SDS-PAGE (10% discontinuous gels, 80 µg proteinwell). A pre-stained standard was used to estimate molecular sizes (MW standard, panels **A** and **B**). Membranes were cut to strips after blocking and incubated separately with human serum samples (indicated in panel **A**) at a dilution of 1:1000. Strips were then fitted together and bound human IgG and IgM (shown in panel **A**) was detected by peroxidase labelled secondary antibodies. Densitometry was performed on the recorded images by ImageJ software, to yield density plots (shown in panels **B**–**E** by dashed (control serum) or continuous (COVID-19 patients’ sera) lines). Pre-stained standard proteins were used to construct a calibration curve (panel **B** lower graph), and molecular size of autoantigens was interpolated according to this calibration curve by GraphPad Prism software. Bands not apparent on the strip incubated by control serum were considered to represent anti-cardiac autoantibody binding to cardiac antigens and are indicated in panels **C**–**E**

Blot images were processed by ImageJ to yield a line-scan of optical densities (Fig. 1A, B). These optical densities were plotted as the function of running distance from the top of the separating SDS–polyacrylamide gel on the membrane (Fig. 1B). Then a calibration curve was produced for each membrane using pre-stained molecular weight standards (Fig. 1B, ProSieve QuadColor protein marker, 4.6–300 kDa, Lonza, Basel, Switzerland). Bands representing cardiac autoantigens were processed similarly (Fig. 1C), and their molecular mass was determined by using the standard curve determined for the same membrane (Fig. 1B). All plotting and interpolations were done by GraphPad Prism software.

### Statistical analysis

**Fig. 2:**
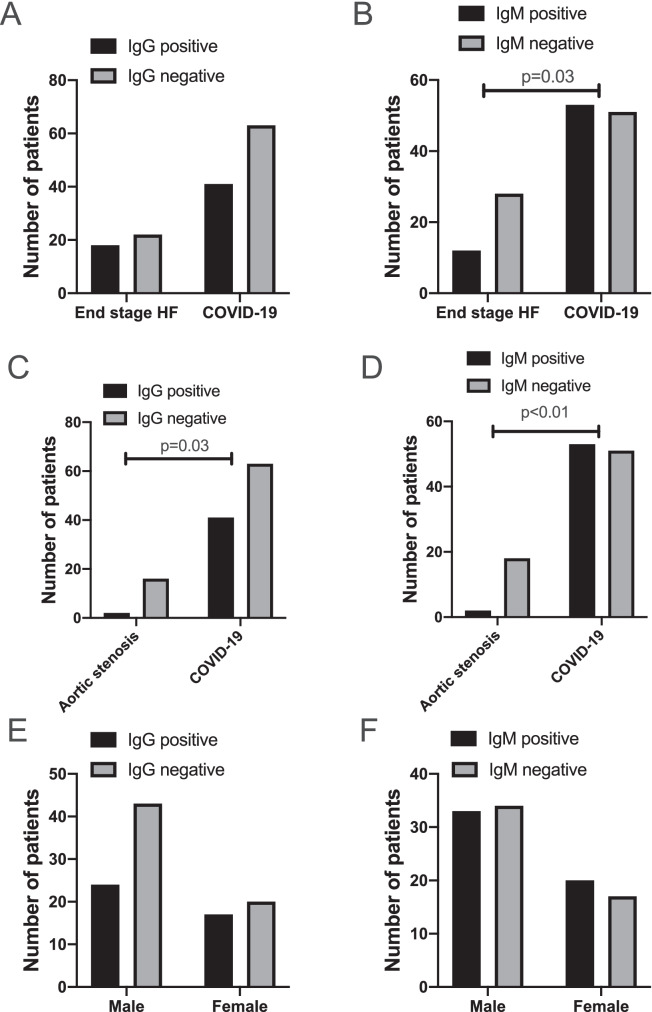
Anti-cardiac autoantibodies in COVID-19 and heart failure patients. Patients were tested for IgG (panels **A**, **C**, and **E**) and IgM (panels **B**, **D**, and **F**) autoantibodies recognizing cardiac proteins by a Western blot-based method. Bars show the number of patients with (black) or without (grey) anti-cardiac autoantibodies. Statistical differences in the occurrence of autoantibodies were tested by Fisher’s exact test and significant differences are indicated (*p* values are also shown). Patient populations included patients with COVID-19 (all panels), patients with endheart failure with dilated cardiomyopathy (panels **A** and **B**), and patients with advanced aortic stenosis (panels **C** and **D**). Potential gender effects in COVID-19 patients were also tested (panels **E** and **F**)

**Fig. 3:**
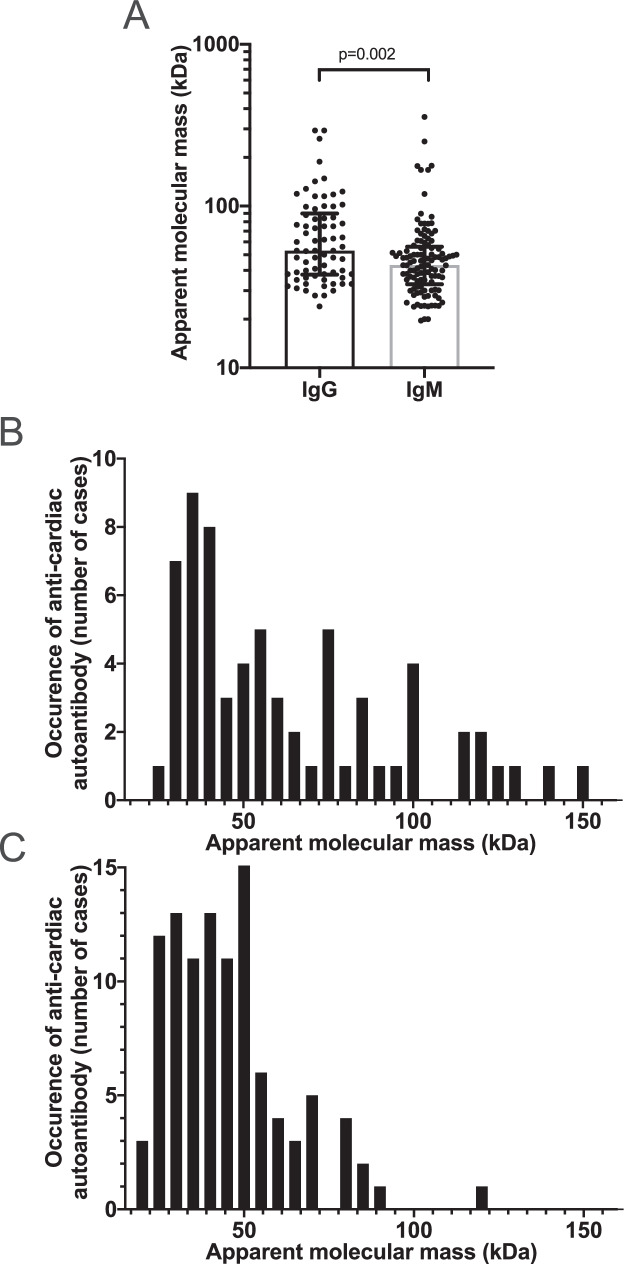
Distribution of cardiac autoantigens. Human cardiac proteins recognized by autoantibodies in the patients’ serum were evaluated here according to their apparent molecular weight. Panel **A** shows all recognized autoantigens. Each symbol represents a band recognized by IgG or IgM type autoantibodies. Bars are median, and error bars represent IQR. Statistical difference is indicated (determined by Mann–Whitney test). Distribution of autoantigens is shown as detected by IgG (panel **B**) and IgM (panel **C**) autoantibodies

Statistical analyses were done by GraphPad Prism software. Mann–Whitney test was used for Fig. 2A, chi square test on Fig. 7A and B, Kruskal–Wallis test was applied on Fig. 7D, Fisher’s exact test was used for Fig. 3A and all comparisons between patients’ populations, Spearman correlation for Fig. 3B, and ROC analysis for Fig. 3A, C. Two-tail tests were done and differences were considered significant if *p* < 0.05.

## Results

COVID-19 patients (*n* = 104) were recruited to test the appearance of anti-cardiac antibodies. The majority of patients were hospitalized (*n* = 97), from which *n* = 58 subjects died. The general clinical characteristics are shown in Table 1.

Presence of both IgG and IgM autoantibodies were tested. More than two-thirds of COVID-19 patients (68%, 71 out of 104) harbored anti-cardiac autoantibodies. There were 39% of patients (41 out of 104) with anti-cardiac IgG autoantibodies (Figs. 1, 2A, 2C, 2E, and all original blots available online), while 51% (53 out of 104) patients harbored anti-cardiac autoantibodies of IgM isotype (Figs. 2B, 2D, 2F, and original blots available online). Multiple cardiac antigens were targeted in 38% (40 out of 104) of COVID-19 patients.

Two control groups were contrasted with the COVID-19 patients. One cohort consisting of 40 individuals represented heart failure patients with dilated cardiomyopathy. We found similar occurrence of anti-cardiac IgG (45%, not significant, Fig. 2A), while significantly lower incidence of IgM (30%, *p* = 0.03, Fig. 2B) autoantibodies in heart failure patients without COVID-19. There were significantly lower occurrence of both IgG (11%, *p* = 0.03, Fig. 2C) and IgM (10%, *p* < 0.01, Fig. 2D) anti-cardiac autoantibodies in patients with advanced aortic stenosis undergoing trans-catheter valve replacement therapy. There was no gender difference among COVID-19 patients in relation to anti-cardiac autoantibody development (Fig. 2E and 2F).

Multiple cardiac antigens were recognized by IgG (12.5%, 13 out of 104) and IgM autoantibodies (26%, 27 out of 104) in COVID-19 patients. Autoantibodies were developed against various human cardiac proteins (Fig. 3A). There was no apparent trend for IgG and IgM type of autoantibodies to recognize a specific myocardial protein (Fig. 3B and 3C and all original blots available online). Instead, a diverse pattern of autoantigens were detected, albeit IgM autoantibodies recognized relatively lower molecular weight autoantigens (*p* = 0.002, Fig. 3A), and were more limited to a set of myocardial antigens.

**Fig. 4:**
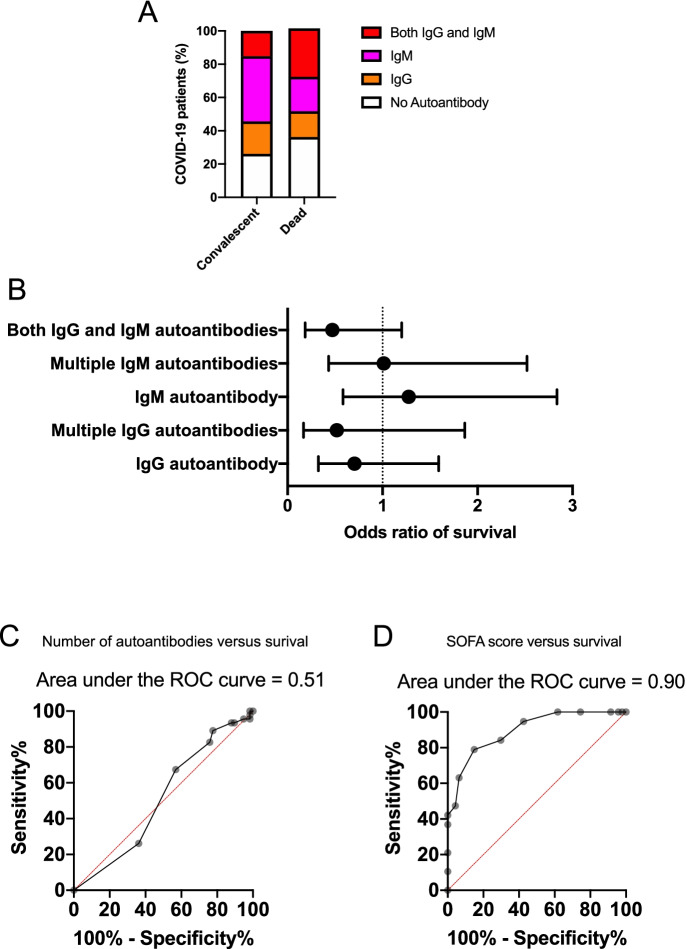
Anti-cardiac autoantibodies do not contribute to acute mortality in COVID-19. Ratios of patients with both IgG and IgM (red), with IgM (magenta), with IgG (orange), and without autoantibodies (empty) are shown on bar graphs in panel **A**. The two bars represent the convalescent and deceased patient populations. Odds ratios were calculated (Fisher’s exact test) for survival of patients according to their autoantibody profile (panel **B**). The potential predictive value of the number of cardiac antigens recognized by autoantibodies in the patients’ sera (panel **C**) and the sequential organ failure (SOFA) score (panel **D**) was tested by ROC analysis. The results of the analyses are shown above the graphs

The presence of anti-cardiac autoantibodies had no apparent contribution to disease severity. There was no difference in the pattern of autoantibody profile between convalescent and deceased individuals (Fig. 4A). The odds ratios showed no significant effects of anti-cardiac autoantibodies on the survival (Fig. 4B). The number of anti-cardiac autoantibodies did not predict mortality (ROC-AUC value: 0.51, *p* = 0.85, Fig. 4C). For comparison, SOFA score predicted mortality in the same patient’s population (ROC-AUC value: 0.90, *p* < 0.01, Fig. 4D).

**Fig. 5:**
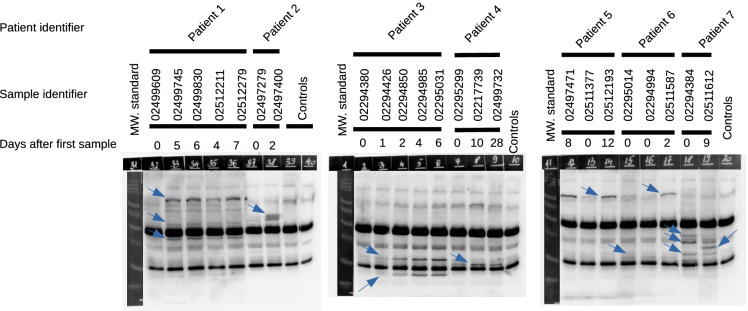
Sequence of resident IgG reactivation in severe COVID-19. In 7 out of 29 cases, we detected changes in the anti-cardiac autoantibody profile during in-hospital treatment of severe COVID-19 patients. Identifiers of the serum samples (8 digit numbers) are shown above the blots. Below the identifiers, the days after the first blood sampling (in-hospital treatment) are shown. Note, samples were ordered on the gels according to identifier number, which was not chronological in some cases. Differences in anti-cardiac autoantibody profiles are indicated by arrows superposed on the immunoblots. Controls represented healthy individuals, without SARS-CoV-2 infection

Some of the patients spent long enough time in the clinical ward to collect sequential blood samples. Activation of resident anti-cardiac autoantibody (IgG type) production was observed during the in-hospital care in a significant number of cases (Fig. 5, patient nos. 1–7). Among the 7 patients, one even showed a significant change in the autoantibody pattern. Some of the autoantibodies weakened, while a new band appeared 9 days later of the previous blood sampling (Fig. 5, patient no. 7). Reactivation of a resident anti-cardiac autoantibody (e.g., type IgG) took about 2–4 days as seen in the individual cases (Fig. 5, patient nos. 1, 2, 3, and 6).

**Fig. 6:**
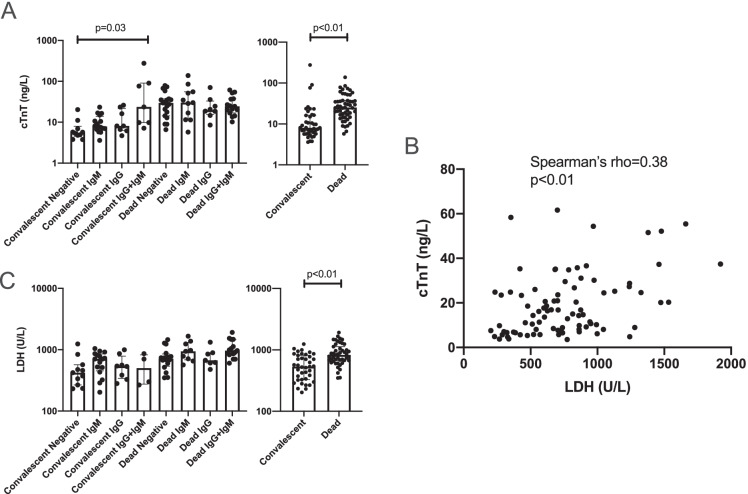
Tissue damage in COVID-19 patients. Clinical parameters of tissue damage were plotted in COVID-19 patients. Cardiac damage was assessed by cardiac troponin T (cTnT) levels in the sera (panels **A** and **B**). Tissue damage was estimated by circulating lactate dehydrogenase (LDH) levels (panels **B** and **C**). Differences among the groups of patients were tested by Kruskal–Wallis test (when multiple groups were compared) or by Mann–Whitney test (pairwise comparisons). Correlation between cTnT and LDH levels was tested by Spearman’s test. Significant differences are indicated with *p* values on the graphs

COVID-19 resulted in a significant damage of myocardial cells. It resulted in the disruption of myocardial integrity and troponin release (Fig. 6A). Note, the level of cardiac troponin T was very high in both convalescent and deceased populations, although it was significantly higher in the later deceased individuals (Fig. 6A). The cardiac protein release can be attributed to general tissue damage in COVID-19, as the troponin T levels in the serum correlated with lactate dehydrogenase (LDH) levels (rho = 0.38, *p* < 0.01, Fig. 6B). LDH levels were generally elevated (Fig. 6C), and somewhat higher in more severe COVID-19 cases.

**Fig. 7:**
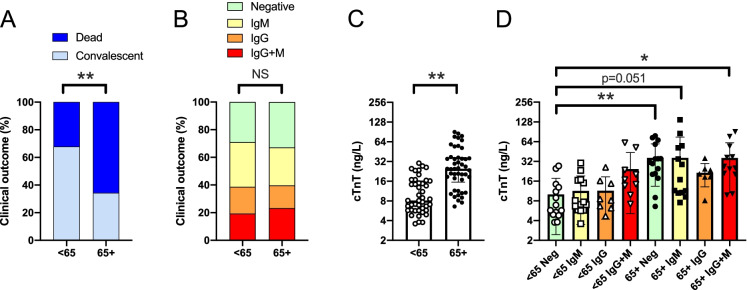
No correlation between aging and anti-cardiac autoantibody production. Patients were stratified as a function of age, generating a younger (age < 65) and an older cohort (age 65 +). In these cohorts, **A** the proportion of the convalescent and fatal outcomes; **B** the relative frequency of autoantibody production; **C** serum cTnT levels are shown; **D** troponin T levels in autoantibody negative, IgM positive, IgG positive, and IgG + IgM positive patients in the two age groups are also plotted

Next, we stratified patients as a function of age that yielded two cohorts, patients below and over 65 years of age (< 65 and 65 + , respectively). In good agreement with prior observations [1]⁠, the fatal outcome of the disease was more common among the elderly (65 + cohort) as compared to the younger (< 65) cohort (*p* < 0.01, Fig. 7A). However, we have not observed higher proportion of autoantibody positive patients among the 65 + patients than among the < 65 patients (Fig. 7B). We observed similar changes when assessing serum cTnT levels, namely, the elderly cohort had higher cTnT levels than the younger cohort (*p* < 0.001, Fig. 7C). Furthermore, cTnT levels did not show statistically significant differences when the patients were stratified as a function of age and type of autoantibody (Fig. 7D).

**Fig. 8:**
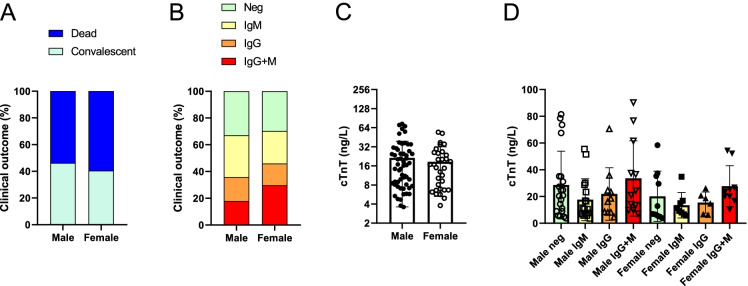
No correlation between gender and anti-cardiac autoantibody production. Patients were stratified as a function of gender. In these cohorts, **A** the proportion of the convalescent and fatal outcomes; **B** the relative frequency of autoantibody production; **C** serum cTnT levels are shown; **D** troponin T levels in autoantibody negative, IgM positive, IgG positive, and IgG + IgM positive patients in the two age groups are also plotted

Finally, we stratified patients as a function of gender. The potential effects of gender were tested on anti-cardiac autoantibody production. There was no difference in morbidity, immunoglobulin production, or cardiac damage between sexes (Fig. 8A-D).

## Discussion

Here we report that the majority of severe COVID-19 patients develop anti-cardiac autoantibodies. Some of these anti-cardiac autoantibodies are of IgM type, suggesting a COVID-19 associated activation of novel autoantibody production (in 63 cases out of total 104). Moreover, frequent appearance of IgG type of anti-cardiac autoantibodies (many cases in addition to IgM) suggest the reactivation of resident anti-cardiac autoantibodies (in 41 out of 104 cases). These findings suggest that acute phase of COVID-19 may be complicated by cardiac autoimmune reactions. Furthermore, dormant autoreactive B cell clones can be reactivated contributing to the autoimmune reaction associated with COVID-19.

A Western blot-based semiquantitative method was used to test anti-cardiac autoantibodies here. Human heart homogenate was used as a bait to pick up potential autoantibodies, recognizing cardiac proteins. This provided a screening assay for cardiac autoantigens, since all recognized proteins were present in the initial heart sample. It also targeted the full human heart proteome, since heart sample was directly homogenized in SDS sample buffer, which can solubilize most of the cardiac proteins. Accordingly, the detected immunoreactivity is a proof of host-targeting immunoglobulins, referred as “anti-cardiac autoantibodies”.

According to our knowledge, development of autoantibodies in COVID-19 patients was first proposed in association with systemic sclerosis [15]⁠, systemic lupus erythematosus (SLE) [16]⁠, and arthritis [7]⁠. These autoantibodies provided a potential cause for symptoms observed in COVID-19 patients. This selected set of autoantibodies was then extended to a full set of extracellular antigens, confirming that autoantibody production is nonspecifically elevated in COVID-19 patients [10]⁠. It was postulated that as much as 20% of death may be related to autoantibodies, mainly developed against IFN [17]⁠. Here we did not attempt to identify and differentiate among the detected antigens targeted by autoantibodies; nevertheless, the large variability in the apparent molecular weight of the bands suggest multiple targets besides to potentially IFN.

There is a limited set of reports on the effects of autoantibody production in the acute phase of COVID-19. An attractive proposal was made by examining 987 critically ill COVID-19 patients, finding that 101 of them had autoantibodies against interferons (IFN), suggesting that autoantibodies may neutralize IFN-mediated signalling [8]⁠. A more recent report confirmed this finding and claimed that blocking of IFN signalling results in a more severe form of COVID-19 based on a detailed analysis of 21 COVID-19 patients [9]⁠.

To the best of our knowledge, this is the first analysis on the potential effect of anti-cardiac autoantibodies in COVID-19. There was no significant effect of anti-cardiac autoantibodies on acute disease mortality in this study. Having a closer look on the odds ratios, it can be noted that the presence of IgM autoantibodies did not affect survival, while the presence of IgG type autoantibodies tends to result in unfavorable outcome. Age or gender had no impact on the prevalence of autoantibody production. The fact that age (in which we expected a gradual decline in immune response) did not affect anti-cardiac autoantibody production can be explained by the enormous inflammatory burst in patients. The case is that these patients do not necessarily represent the general younger and elderly population. In contrast, these patients represent critically ill (patients having super-normal inflammatory burst in response to SARS-CoV-2 infection) individuals from each age group. The question of the modulatory role of age on the ability to develop hyper-inflammation is out of the scope of this manuscript.

It was proposed that post-acute sequelae of COVID-19 (PASC) can be attributed to four main risk factors at the time of initial COVID-19 diagnosis: type 2 diabetes mellitus, SARS-CoV-2 RNAemia, Epstein-Barr virus viremia, and specific autoantibodies [17]⁠. In the light of the findings of this study, we propose that anti-cardiac autoantibodies may contribute to cardiac complications in long COVID, or PASC. As a matter of fact, cardiovascular complications are frequently present in acute COVID-19 [18]⁠ and may be a significant factor after the acute phase of COVID-19 [3–5, 19]⁠. Among the proposed post-COVID symptoms, anti-cardiac autoantibodies may contribute to myocarditis, dilated cardiomyopathy [20]⁠, or cardiac arrhythmias [21]⁠. Indeed, patients with dilated cardiomyopathy (recruited before COVID-19 pandemic) tested in this report had similarly high number of IgG autoantibodies than that developed in COVID-19 patients. This correlation needs to be tested by future, longitudinal studies to confirm a causative relationship.

Anti-cardiac autoantibody development in COVID-19 patients was compared to two severe cardiac patients’ populations. Anti-cardiac autoantibody (IgG and IgM) production was very low (significantly lower than that in COVID-19 patients) in patients with severe aortic stenosis, undergoing trans-catheter valve implantation. In contrast, patients with advanced heart failure with dilated cardiomyopathy had similar occurrence of IgG and lower occurrence of IgM anti-cardiac immunoglobulins. This suggests that cardiac failure (similarly to severe COVID-19) results in the release of immunogenic cardiac proteins, which may lead to reactivation of anti-cardiac autoantibodies. The apparent difference between anti-cardiac IgG antibodies in the two heart failure populations (with dilated cardiomyopathy or with aortic stenosis) suggests that differences in the pathomechanism of these diseases may have a decisive role in autoimmunity. We hypothesize that severe COVID-19 patients suffered from multiorgan failure, including cardiac failure, making them susceptible for cardiomyocyte damage. Note that anti-cardiac IgM autoantibodies were significantly higher in COVID-19 patients, suggesting that the pro-inflammatory state can induce the development of novel anti-cardiac autoantibodies. This was observed in more than half of the cases, suggesting a potential long-term impact of these novel autoantibodies.

This report also shed some light on the kinetics of reactivation of anti-cardiac autoantibody production. We observed a significant increase in IgG autoantibodies within a 2-day period. It suggests that reactivation of IgG autoantibodies can occur in 2 days. Note, appearance of IgG antibodies can also be explained by seroconversion from newly developed IgM antibodies to IgG isotype. However, we did not observe similar autoantigen pattern in the same patients when tested for IgM autoantibodies. In the case of IgM autoantibodies, we did not attempt to track the kinetics. Nonetheless, the fact that almost two-thirds of the patients had some level of IgM autoantibody recognizing cardiac proteins suggests that severe COVID-19 is a powerful inducer of heart-targeting autoantibody production. It is probably linked not only to myocardial damage but also to the powerful activation of the immune system, resulting in cytokine storm in many severe COVID-19 patients. Taken together, severe COVID-19 may induce novel autoantibodies (as reflected by IgM autoantibodies) and also reactivate resident autoantibodies (as reflected by IgG autoantibodies). This is probably mediated by both inflammatory conditions (as a response to viral infection) and cardiac protein release (cardiac injury) to the bloodstream. It appears that the autoimmune reaction can be proportional with the severity of COVID-19 and also with the anti-viral antibody production [22]⁠. It is also important to note that IgM to IgG seroconversion can occur relatively rapidly in COVID-19 patients, suggesting that some of the detected IgG autoantibodies can be the result of SARS-CoV-2 infection [22]⁠.

Finally, this report suggests that anti-cardiac autoantibodies contribute to the multisystem inflammatory syndrome (MIS), which often complicates SARS-CoV-2 infections [6]⁠. The abundance of anti-cardiac autoantibodies reported here provides a link to cardiac damaging autoimmunity in MIS-C [23]⁠ and MIS-A [6]⁠.

This study has many limitations. One of the major limitations is that although the detection method is specific for human (host) cardiac proteins, it is only semiquantitative (at best) regarding the titer of the autoantibodies. The intensity of the bands varied greatly in the study, and the authors opted to not differentiate between the autoantibodies on this basis. Another limitation is that the size of the targeted myocardial proteins was determined by densitometry, resulting only in an approximate size. The scatter resulting from this determination can be judged by the fact that IgG heavy chain showed a molecular weight of 47.9 ± 3.1 kDa, while the light chain gave 27.5 ± 1.9 kDa size (mean ± SD, *n* = 35). The results represent the retrospective evaluation of a mainly Caucasian population. Finally, the patient recruitment was done in the second wave of the COVID-19 in Hungary, most probably representing patients infected by the original Wuhan strain of SARS-CoV-2.

## Supplementary Information

Below is the link to the electronic supplementary material.

Supplementary file1 (PDF 293 KB)
