# Supplementary material for: The majority of severe COVID-19 patients develop anti-cardiac autoantibodies
Source: GeroScience. 2022 Sep 16;44(5):2347–60. doi: 10.1007/s11357-022-00649-6 (PMC9483490; doi:10.1007/s11357-022-00649-6)

Fig. s1

Loading MW standard (single well) or human heart homogenate (all other wells)

SDS-PAGE (10% gel)

Transfer onto nitrocellulose

Cutting membrane to strips (aided by Ponceau staining)

Blocking nonspecific binding sites (non-fat milk 5%)

Placing strips into individual trays for separate incubations

Incubation with human Serum (1:1,000)

Collecting strips into a single tray for further incubations

Incubation with Peroxidase labelled anti-human IgG (1:25,000) or IgM (1:10,000)

Placing strips to reconstitute original membrane

Enhanced Chemiluminescence (ECL) reaction

Recording by digital camera

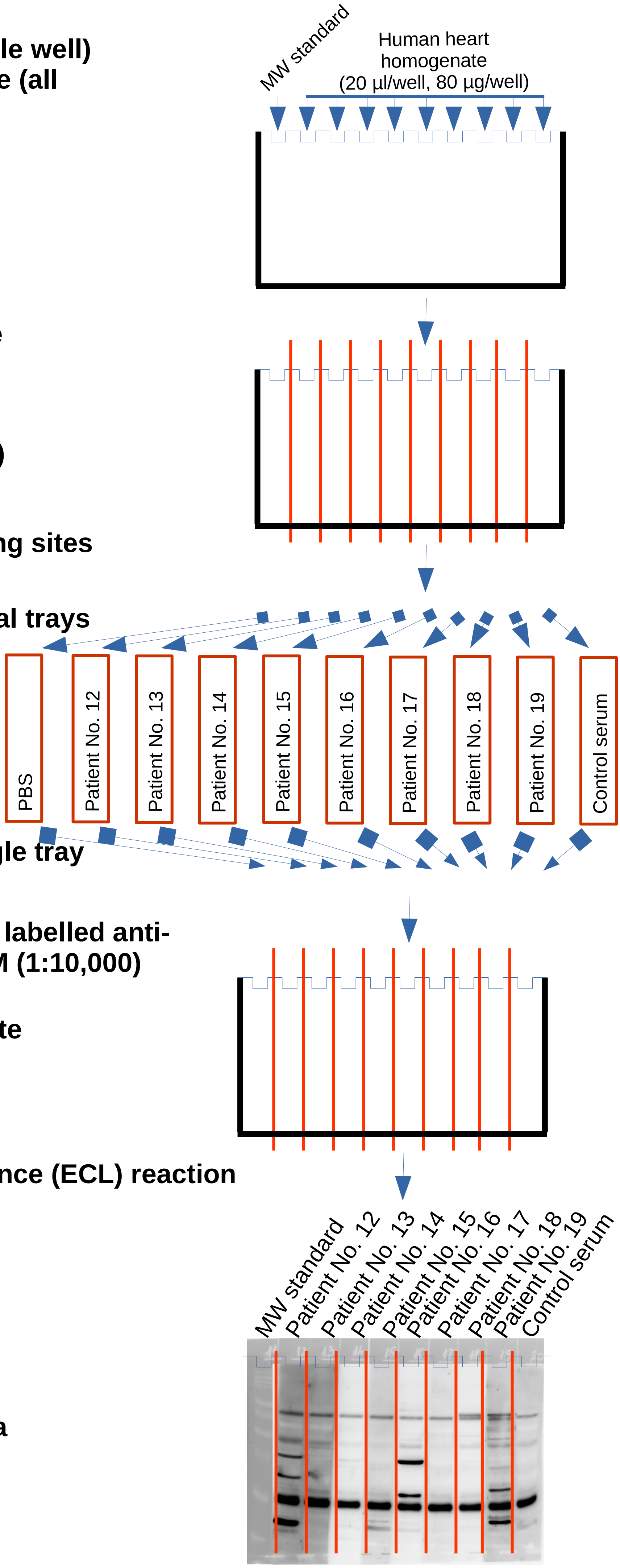

Supplement: Supplementary file 1 — Supplementary file1 (PDF 293 KB) [file 11357_2022_649_MOESM1_ESM.pdf]
